# Azathioprine-Induced Eosinophilia and Acute Cholestatic Hepatitis in Lupus Nephritis: An Early Hypersensitivity-Mediated Presentation of Thiopurine-Associated Drug-Induced Liver Injury

**DOI:** 10.7759/cureus.105991

**Published:** 2026-03-27

**Authors:** Sara Ali, Kian Memari, Josue Boutros, Shane Williams, Peter Cohen, Lissette P Lazo

**Affiliations:** 1 Medicine, Nova Southeastern University Dr. Kiran C. Patel College of Osteopathic Medicine, Fort Lauderdale, USA; 2 Family Medicine, Palmetto General Hospital, Hialeah, USA; 3 Family Medicine, Nova Southeastern University Dr. Kiran C. Patel College of Osteopathic Medicine, Fort Lauderdale, USA

**Keywords:** azathioprine, drug-induced liver injury, eosinophilia, hepatotoxicity, immunosuppressive therapy, lupus nephritis

## Abstract

Azathioprine is a thiopurine immunosuppressive medication widely used in the treatment of autoimmune diseases and organ transplantation. Although hematologic toxicity is well recognized, hepatotoxicity and hypersensitivity reactions are less commonly encountered but clinically significant adverse effects.

We present the case of a 20-year-old female with systemic lupus erythematosus and class III lupus nephritis who developed progressive nausea, fatigue, eosinophilia, pancytopenia, and marked transaminase elevation shortly after initiation of azathioprine therapy. Imaging excluded biliary obstruction, while endoscopic and histologic evaluation demonstrated inflammatory changes with eosinophilic infiltration. Liver biopsy confirmed acute intrahepatocellular cholestatic hepatitis.

Discontinuation of azathioprine led to rapid biochemical improvement and resolution of eosinophilia. This case highlights an early hypersensitivity-mediated presentation of azathioprine-induced liver injury and underscores the importance of early recognition and prompt drug withdrawal.

## Introduction

Azathioprine is a widely used thiopurine immunosuppressive medication employed in the management of autoimmune diseases, including systemic lupus erythematosus, inflammatory bowel disease, and rheumatoid arthritis, as well as in organ transplant maintenance therapy [1]. As a prodrug of 6-mercaptopurine, azathioprine interferes with purine synthesis and inhibits the proliferation of rapidly dividing lymphocytes [1].

The most frequently reported adverse effects involve bone marrow suppression, particularly in patients with thiopurine methyltransferase (TPMT) deficiency or altered drug metabolism [2]. In addition to hematologic toxicity, azathioprine has been associated with hepatotoxicity, although this occurs less commonly and may be underrecognized in clinical practice [3].

Azathioprine-induced hepatotoxicity may present as transient transaminase elevation, cholestatic hepatitis, veno-occlusive disease, or nodular regenerative hyperplasia [3]. While most cases occur after several months of therapy, early-onset presentations have been described, particularly in the setting of hypersensitivity reactions [4].

Peripheral eosinophilia is a rare manifestation of thiopurine therapy and may reflect an immune-mediated response. The coexistence of eosinophilia and hepatotoxicity should raise concern for drug-induced liver injury and prompt immediate evaluation and discontinuation of the offending agent.

## Case presentation

A 20-year-old female with systemic lupus erythematosus complicated by class III lupus nephritis presented with progressive nausea, weakness, and fatigue over one month.

Initial laboratory evaluation demonstrated pancytopenia with relative eosinophilia and marked hepatic enzyme elevation. Alanine aminotransferase was 330 U/L, aspartate aminotransferase 750 U/L, gamma-glutamyl transferase 819 U/L, and total bilirubin 2.1 mg/dL. Alkaline phosphatase was elevated at 300 U/L (Table 1).

**Table 1:** Detailed laboratory investigations µL: microliter; mm³: cubic millimeter; gm/dL: grams per deciliter; fL: femtoliter; pg: picogram; mmol/L: millimoles per liter; mg/dL: milligrams per deciliter; mcg/dL: micrograms per deciliter; U/L: units per liter;  mL/min/1.73 m²: milliliters per minute per 1.73 square meters of body surface area; AST: aspartate aminotransferase; ALT: alanine aminotransferase; GGT: gamma-glutamyl transferase; WNL: within normal limits

Parameter (Unit)	Patient’s Value	Normal Reference Range	Clinical Note/Interpretation
White Blood Cell (µL)	2.1	5.0 - 11.0 x 10^3^	-
Red Blood Cell (mm^3^)	3.0	4.70 - 6.10 x 10^6^	-
Hemoglobin (gm/dL)	8.9	14.0 - 18.0	-
Hematocrit (%)	27.0	42.0 - 52.0	-
Mean Corpuscular Volume (fL)	90	80 - 94	Within Normal Limits (WNL)
Mean Corpuscular Hemoglobin (pg)	31.0	27.0 - 31.0	WNL
Mean Corpuscular Hemoglobin Concentration (gm/dL)	33.0	33.0 - 37.0	WNL
Red Cell Distribution Width - Coefficient of Variation (%)	16.2	11.5 - 14.5	-
Platelet Count (µL)	72	130 - 140 x 10^3^	-
Eosinophils (%)	14	0 - 5	-
Absolute Eosinophil Count (µL)	0.29	0 - 0.5 x 10^3^	-
Sodium (mmol/L)	131	137 - 145	-
Potassium (mmol/L)	3.7	3.4 - 5.0	WNL
Chloride (mmol/L)	96	98 - 107	-
Carbon Dioxide (mmol/L)	24	22 - 30	WNL
Blood Urea Nitrogen (mg/dL)	12	9.0 - 20.0	WNL
Creatinine (mg/dL)	0.82	0.66 - 1.25	WNL
Estimated Glomerular Filtration Rate (mL/min/1.73 m^2^)	104	≥ 90	WNL
Glucose (mg/dL)	142	74.0 - 106.0	-
Calcium (mg/dL)	8.7	8.4 - 10.2	WNL
Phosphorus (mg/dL)	3.3	2.5 - 4.5	WNL
Total Bilirubin (mcg/dL)	2.10	0.20 - 1.30	-
AST (U/L)	750	17 - 59	-
ALT (U/L)	330	21 - 72	-
ALP (U/L)	300	44 - 147	-
GGT (U/L)	819	5 - 36	-
Urinalysis Protein (mg/dL)	>300	<15	-

Abdominal ultrasound demonstrated mild hepatomegaly and gallbladder sludge without biliary dilation. Magnetic resonance cholangiopancreatography revealed no evidence of biliary obstruction.

Esophagogastroduodenoscopy demonstrated a gastric ulcer, and biopsy revealed eosinophilic infiltration of the lamina propria. Liver biopsy demonstrated macrovesicular steatosis and acute intrahepatocellular cholestatic hepatitis.

Azathioprine was discontinued, and the patient was treated with supportive therapy including corticosteroids and proton pump inhibitors.

**Figure 1:**
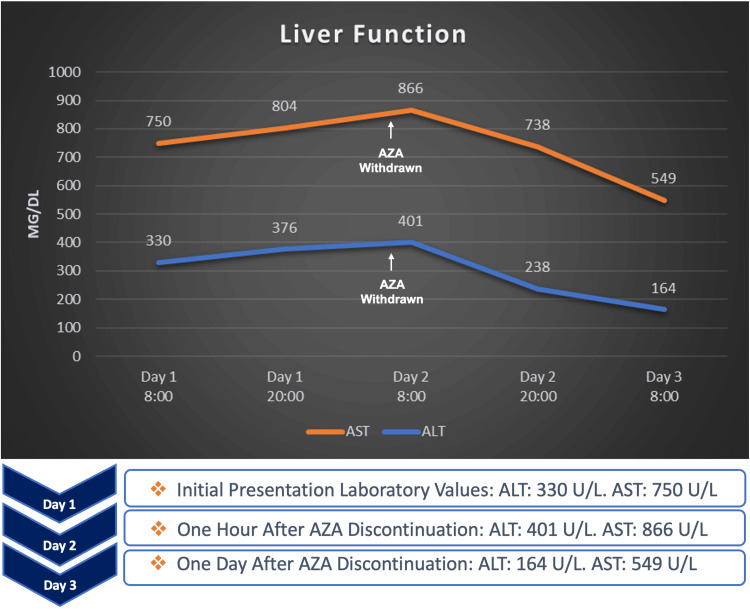
Improvement of Liver Function Tests after Azathioprine Discontinuation Linear graph with accompanying key event highlights illustrating the dynamic trend of aspartate aminotransferase (AST, orange line) and alanine aminotransferase (ALT, blue line) serum levels. The initial measurement (Day 1) demonstrates marked elevations (AST 750 U/L, ALT 330 U/L). A paradoxical rise in enzymes is noted one hour after azathioprine withdrawal on Day 2, with levels peaking at 866 U/L for AST and 401 U/L for ALT. A significant and steady decline is observed by Day 3, with levels decreasing to 549 U/L for AST and 164 U/L for ALT, representing a strong correlation between medication discontinuation and the improvement of liver enzymes. This figure was created by the authors using original data and conceptual illustration prepared for this manuscript. No artificial intelligence (AI) or large language model (LLM) tools were used in the creation or modification of this figure.

Following discontinuation, eosinophilia resolved and liver enzyme levels progressively improved. A transient rise in transaminases was observed shortly after drug cessation before a steady decline. By Day 3, alanine aminotransferase decreased to 164 U/L and aspartate aminotransferase to 549 U/L, demonstrating a rapid initial improvement (Figure 1).

**Figure 2:**
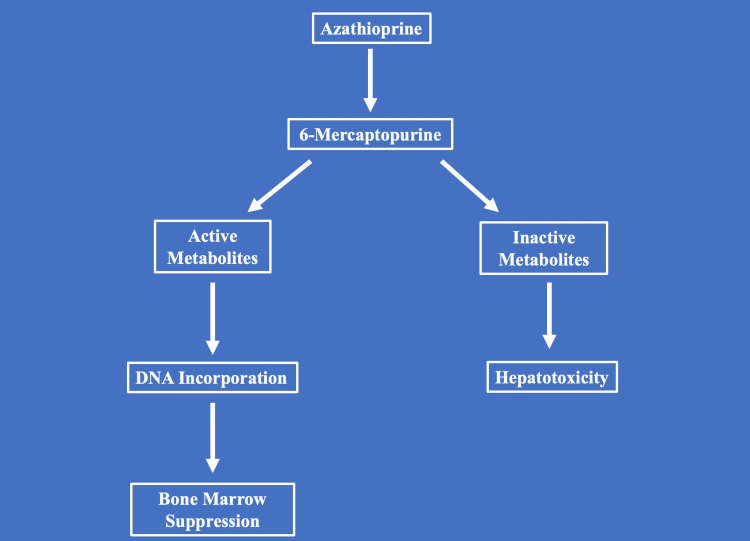
Azathioprine Metabolism This figure was created by the authors using original data and conceptual illustration prepared for this manuscript. No artificial intelligence (AI) or large language model (LLM) tools were used in the creation or modification of this figure.

Azathioprine is converted to 6-mercaptopurine (6-MP), which undergoes metabolism through several competing enzymatic pathways. One pathway leads to the formation of active 6-thioguanine nucleotides (6-TGNs), which incorporate into DNA and are responsible for the drug’s immunosuppressive efficacy but may also contribute to bone marrow suppression. Alternatively, 6-MP can be metabolized by thiopurine methyltransferase (TPMT) to 6-Methyl MP, a metabolite associated with hepatotoxicity. A third pathway involves xanthine oxidase-mediated conversion of 6-MP to thiouric acid, an inactive metabolite. Genetic or enzymatic variations in these metabolic pathways can influence drug efficacy and the risk of adverse effects (Figure 2).

## Discussion

Azathioprine-induced liver injury is an uncommon but clinically significant adverse effect, with reported rates of hepatotoxicity ranging from approximately 1% to 3% of treated patients [3]. Although most cases develop after several months of therapy, early-onset presentations have been described and are often associated with hypersensitivity-mediated mechanisms [4,5]. The present case is notable for the rapid onset of severe transaminitis and eosinophilia within one month of azathioprine initiation, supporting an idiosyncratic immune-mediated process rather than cumulative dose-dependent toxicity.

A key biochemical feature in this case is the pattern of liver injury. In addition to elevated transaminases, the patient demonstrated a markedly elevated gamma-glutamyl transferase and hyperbilirubinemia, suggesting cholestatic involvement. Assuming an alkaline phosphatase (ALP) value of approximately 300 U/L (upper limit of normal ~120 U/L), the calculated R-value would be: R = (ALT/ULN) ÷ (ALP/ULN) = (330/72) ÷ (300/120) ≈ 4.6 ÷ 2.5 ≈ 1.8. This R-value is consistent with a cholestatic pattern of liver injury (R < 2), which aligns with the patient’s clinical presentation, laboratory findings, and biopsy-proven intrahepatocellular cholestatic hepatitis. Cholestatic and mixed patterns are well-described manifestations of thiopurine-associated hepatotoxicity [3].

An additional notable feature of this case is the transient rise in transaminases observed shortly after discontinuation of azathioprine. Although the precise mechanism is not fully understood, this phenomenon may represent a delayed peak in the immune-mediated injury cascade or the release of intracellular enzymes during hepatocyte turnover and apoptosis rather than ongoing hepatotoxic exposure. Recognition of this pattern is important in clinical practice to avoid misinterpretation as treatment failure or disease progression.

The interpretation of eosinophilia in this case also warrants careful consideration. While the eosinophil percentage was elevated at 14%, the absolute eosinophil count remained within normal limits. This discrepancy is explained by concurrent leukopenia, resulting in relative eosinophilia rather than true peripheral eosinophilia. However, the presence of eosinophilic infiltration on gastrointestinal biopsy supports a localized hypersensitivity response, reinforcing an immune-mediated mechanism of injury. Eosinophilia, whether relative or absolute, has been described in association with drug-induced liver injury and may serve as a clinical clue to hypersensitivity-related etiologies [4].

Thiopurine metabolism is influenced by enzymatic pathways including thiopurine methyltransferase (TPMT), and deficiency in TPMT activity has been associated with increased risk of hematologic toxicity [2]. In this case, TPMT activity or genotyping was not performed, representing a limitation. However, the early onset of symptoms and the presence of hypersensitivity features suggest that the observed toxicity is more consistent with an idiosyncratic immune-mediated reaction rather than impaired drug metabolism alone.

Prompt recognition and discontinuation of azathioprine resulted in rapid biochemical improvement, further supporting the diagnosis of drug-induced liver injury. Drug-induced liver injury remains one of the leading causes of acute liver dysfunction in developed countries, and early identification of the offending agent is critical to preventing progression to severe hepatic injury or liver failure [4]. This case underscores the importance of maintaining a high index of suspicion for drug-induced liver injury in patients presenting with unexplained transaminitis, particularly when accompanied by atypical features such as eosinophilia.

## Conclusions

Azathioprine remains an important immunosuppressive therapy; however, clinicians must remain vigilant for rare adverse effects such as drug-induced liver injury and hypersensitivity-associated eosinophilia. Although hepatotoxicity typically develops after prolonged exposure, early-onset presentations may occur. In this case, transaminase levels demonstrated a rapid initial improvement following drug cessation, though values remained elevated early in the recovery phase, emphasizing the importance of continued monitoring.

Early recognition and prompt discontinuation of the offending agent are critical to preventing progression to severe hepatic injury.
